# Integrative Analysis of Small RNA and mRNA Expression Profiles Identifies Signatures Associated With Chronic Epididymitis

**DOI:** 10.3389/fimmu.2022.883803

**Published:** 2022-05-11

**Authors:** Jialei Gong, Peng Wang, Jin-Chuan Liu, Jianlin Li, Qun-Xiong Zeng, Chen Yang, Yanfeng Li, Di Yu, Dandan Cao, Yong-Gang Duan

**Affiliations:** ^1^Shenzhen Key Laboratory of Fertility Regulation, Center of Assisted Reproduction and Embryology, The University of Hong Kong – Shenzhen Hospital, Shenzhen, China; ^2^The University of Queensland Diamantina Institute, Faculty of Medicine, The University of Queensland, Woolloongabba, QLD, Australia; ^3^Department of Urology, Daping Hospital, Army Medical University, Chongqing, China; ^4^Department of Obstetrics and Gynecology, The University of Hong Kong, Hong Kong, Hong Kong SAR, China

**Keywords:** multi-omics, small RNAs, microRNA, tsRNA, mRNA, integrative analysis

## Abstract

Chronic epididymitis (CE) refers to a long-lasting inflammatory condition of the epididymis, which is considered the most common site of intrascrotal inflammation and an important aetiological factor of male infertility. Recent studies demonstrate that small RNAs secreted from epididymal epithelium modulate embryo development and offspring phenotypes *via* sperm transmission, and the resulting modifications may lead to transgenerational inheritance. However, to date, the genome-wide analysis of small RNA together with the transcriptomic expression profiles of human epididymis and CE is still lacking. In this study, we facilitated next-generation sequencing and bioinformatics to comprehensively analyze the small RNA and mRNA in an integrative way and identified signatures associated with CE. Both of the small RNA and mRNA expression data demonstrated relatively larger molecular differences among the segmental region of the epididymides, including caput, corpus, and cauda, than that of the inflammatory conditions. By comparing the inflamed caputs to the controls, a total of 1727 genes (1220 upregulated and 507 downregulated; 42 most significant genes, adjusted *P <*0.05) and 34 miRNAs (23 upregulated and 11 downregulated) were identified as differentially expressed. In silico functional enrichment analysis showed their roles in regulating different biological activities, including leukocyte chemotaxis, extracellular milieu reconstruction, ion channel and transporter-related processes, and nervous system development. Integrative analysis of miRNA and mRNA identified a regulatory network consisting of 22 miRNAs and 31 genes (miRNA-mRNA) which are strong candidates for CE. In addition, analysis about other species of small RNA, including (miRNA), piwi-interacting RNA (piRNA), tRNA-derived small RNA (tsRNA), Y RNA, and rsRNA identified the distinct expression pattern of tsRNA in CE. In summary, our study performed small RNA and miRNA profiling and integrative analysis in human CE. The findings will help to understand the role of miRNA-mRNA in the pathogenesis of CE and provide molecular candidates for the development of potential biomarkers for human CE.

## Introduction

Chronic epididymitis (CE) is a long-lasting inflammatory condition of the epididymis, which is considered the most common site of intrascrotal inflammation (1, 2). Since infection and inflammation of the genital tract are thought to be a primary aetiological factor of male infertility, CE appears to be more critical than prostatitis or seminal vesiculitis due to the direct interaction between sperm cells and epididymal epithelium. A previous study demonstrates that up to 40% of patients with oligozoospermia and azoospermia are affected by CE (3). Prevalence rates of 6%-15% have been reported from andrological outpatient clinics (4, 5), and it is the most cause of scrotal pain in adults, reaching up to 600 000 cases/yr in the US (6).

Previous studies demonstrate that region-specific microRNA (miRNA) profiles have been characterized in rodents and human epididymis (7, 8). MiRNA could be released from the epididymal epithelium in exosomes, which can be taken up by transiting sperm (9). Recent studies suggest sperm tRNA-derived small RNAs (tsRNA) modulate embryonic development and offspring phenotypes (10, 11). These small RNAs of spermatozoa secreted from the epididymis are thought to be essential for embryonic development. However, the expression profiles of small RNAs and mRNA in inflamed epididymis have received little attention in spite of the fact that they might play a key role in immune regulation and epigenetic transmission. In chronic inflammation, epigenetics is thoroughly involved in the behavior regulation of immune cells because they convert the information from the infection environment to responsive intracellular signals by small molecules, including metabolic intermediate (12). In a lipopolysaccharide (LPS)-induced epididymitis of rat model, a total of 1378 differentially expressed genes was identified compared with the controls through RNA sequencing, including 531 upregulated and 847 downregulated genes (13). Furthermore, a recent study stressed the critical function of tsRNA in transgenerational transmission induced by epididymal inflammation (14), the epigenetic contribution of inflammation in CE, particularly of small RNA and mRNA modifications, remains largely unexplored. Therefore, our study aims to analyze the small RNA landscape and transcriptional characteristics in human CE, as well as the potential correlation between them. The study provides a potential basis to understand the cause and potential molecular events of CE, prospectively offering more specific and effective molecular targets for the treatment of CE and male infertility.

## Materials and Methods

### Patient Samples and RNA Extraction

Epididymal biopsies from adult men undergoing urological work-up were retrieved from the archive. The patients who underwent epididymectomy for CE between 2018 and 2020 at our institution were identified. Inclusion criteria were the previous history of bacterial epididymitis, increased numbers of macrophages and/or dendritic cells in semen, clinical symptoms of continuous epididymal pain for more than six weeks and diagnosis of chronic epididymitis by scrotal ultrasonography, no pain relief after six weeks of conservative antibiotics and anti-inflammatory drug treatment, and willingness to receive epididymectomy. The exclusion criteria were urinary tract infection, prostatitis, chronic pelvic pain syndrome, previous vasectomy, previous scrotal surgery, and the presence of concurrent diseases such as an epididymal cyst or a granuloma, both of which can cause scrotal pain. According to the histopathological evaluation, patients with both impaired epididymal structure and signs of inflammation (as defined by the occurrence of distinct round cells infiltrating perivascularly or interstitially) were included in the study (*n* = 5). For comparison, the controls were collected from patients who underwent testectomy for castration therapy of prostate cancer. The specimens revealing the normal epididymal structure and no other obvious pathological alterations were selected as controls (*n* = 5). Patient characteristics were summarized in **Supplemental Table S1**. All samples were obtained after getting the written informed consent from all participants. The study was conducted with the approval of the Institutional Review Board of the hospital (HKU-SZH_IRB_ [2017] _22) according to the principles expressed in the Declaration of Helsinki.

Samples were frozen in liquid nitrogen, and the total RNAs were extracted using Trizol (Invitrogen) according to the manufacturer’s protocol. RNA quality was confirmed by Nanodrop measurement of OD 260/280 and 260/230 ratios, and RNA integrity was examined by Agilent 2100 BioAnalyzer.

### RNA Sequencing

For each sample, 1 μg of total RNA was used for cDNA library preparation, employing the TruSeq RNA Sample Preparation Kit v2 as per the manufactory protocol. The cDNA library was then sequenced in a pair-end 100bp mode on Hiseq 2500 platform (Illumina).

### Small RNA Sequencing

A total of 3ug RNA of each sample was used for small RNA library preparation, employing NEBNext^®^ Multiplex Small RNA Library Prep Set for Illumina^®^as per the manufactory protocol. The purified small RNA libraries were then quantified with QubitFluorometer (Invitrogen) and used for cluster generation through Cluster Generation (cBot, Illumina). Single-end 36bp sequencing was run on HiSeq2500 (Illumina) according to the manufacturer’s instructions.

### mRNA Profiling

The raw mRNA reads undergo quality control, and low-quality reads cleaning through Fastqc and Fastp, two packages for sequence pre-disposal. After which, reads were aligned to the GRCh38 reference genome by mapping alignment software hisat2 2.2.2 and counted through FeatureCounts 2.2.0. Pre- and Post-alignment QC was determined on each sequenced sample. Aligned Bam files were subjected to quantification using FeatureCounts.

### Small RNA Profiling

The raw small RNA reads undergo quality control and cleaning as the same as mRNA reads. The clean reads were aligned to the GRCh38 reference genome through Bowtie. miRNA was annotated and quantified using miRDeep2, pl, while tsRNAs and piRNAs were annotated and quantified using SPORT.

### Dimensional Reduction Analysis

The two-dimensional reduction analysis PCA and UMAP performed on normalized gene expression value were implemented in R by stats 4.0.3 and umap 0.2.6.0. PCA parameter was set as n_components=2, scale=T; UMAP parameter was set as n_components=2, n_neighbors=2, min_dist=0.1, metric=‘euclidean’, n_jobs=-1.

### Differentially Expressed Genes (DEGs) and MiRNAs (DEMs) Analysis

Differentially expressed genes (DEGs) and miRNAs (DEMs) were assessed by the DESeq2 package. Read counts of CE and non-CE samples were firstly normalized through the Relative Log Expression (RLE) model and then subjected to test for DEGs or DEMs using wald-test under a Negative Binomial Distribution modeling. DEGs and DEMs were filtered with the criteria of p-value < 0.05 and |log2 fold change | > 1.

### Functional Enrichment Analysis for DEGs and DEMs

To explore the possible functional roles of the identified DEGs, Gene ontology analysis and KEGG pathway analysis were conducted through online analytic tools DAVID in R. For DEMs, the potential downstream target genes were evaluated through online tools miRWalk. The common predicted target genes for one miRNA by at least two of three tools Target, Mirdb, and Mirtabase, were selected for GO and KEGG enrichment analysis. The Enriched terms with *P*-values<0.05 and FDR <0.05 were selected, and top terms were displayed. Gene set enrichment analyses were also conducted for DEGs and the selected target gene sets of DEMs through GSEA software in Windows, and top enriched gene terms were visualized.

### Integrative Analysis of DEGs and DEMs

The potential target genes of DEMs were intersected with DEGs. Overlapped DEGs were imported into Cytoscape, along with DEMs, to generate the interactive network. The fold change of DEGs and DEMs was annotated to the network by color.

### Validation of DEGs by Real-Time PCR

Six DEGs (GSDMA, CXCL6, FOSB related with inflammation; MUC16, AGTR2, TREM1 associated with fibrosis) were selected for confirmation with real-time PCR. The procedures were similar to those above, and the primer used are listed in **Supplemental Table S5**.

## Results

### High Transcriptional Heterogeneity Across the Segmental Region of the Epididymides

Five CE samples and five control counterparts were collected from CE patients undergoing epididymis excision, including seven caputs, two corpora, and one cauda. The histopathology of the epididymides was assessed in paraffin and the sections were stained with hematoxylin and eosin. The histopathological changes were characterized by mononuclear cell infiltration in the interstitial of the CE samples (**Supplemental Figure S1**). Each sample was then be sequenced to profile the mRNA and small RNA landscape separately. RNA-seq produced 192098872 raw reads from CE samples and 240886956 raw reads from the controls as shown (**Supplemental Table S2**). The proportion of clean reads was >90% for each sample, out of which >95% was mappable to GRCh38 human reference genome achieving a unique map rate >95%.

**Figure 1:**
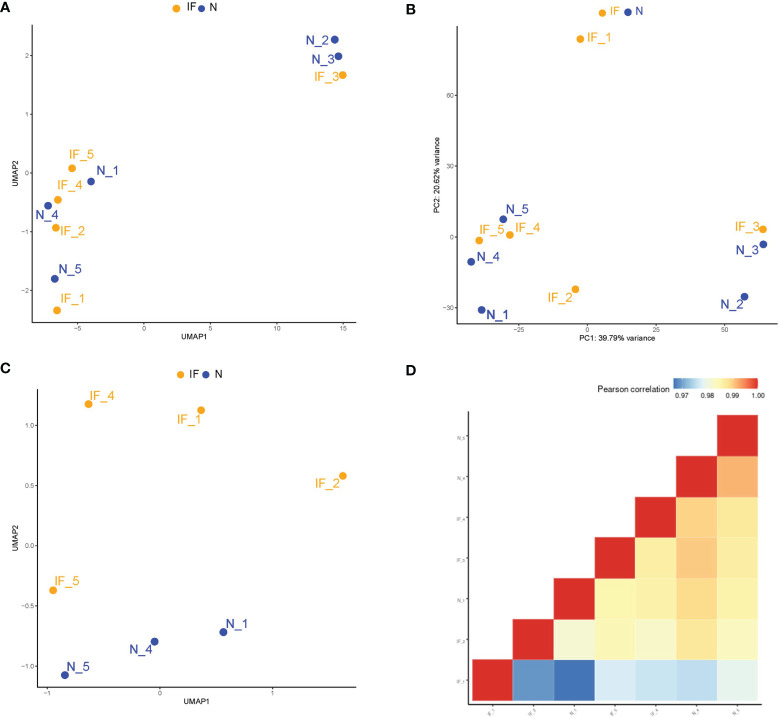
Higher transcriptional heterogeneity acrossed the segmental region than that of the inflammatory conditions in the epididymides. **(A)** Sample distributions in a two-dimension space by UMAP based on global mRNA expression. IF represented the inflamed epididymides, and N indicated the controls. **(B)** Sample distributions in a two-dimension space by the principal components analysis (PCA) based on global mRNA expression. **(C)** Distributions in two-dimension space by UMAP based on global mRNA expression after removing the corpus and cauda samples. **(D)** Correlation matrix of the remaining 7 samples by global mRNA expression of the caput epididymides.

Overall counts of each sample were normalized and standardized for further analysis. The Uniform Manifold Approximation and Projection for Dimension Reduction (UMAP) revealed the cluster structure of all samples (**Figure 1A**). The UMAP results demonstrated that three samples (IF-3, N-2, N-3) from corpus and cauda of the epididymides were segregated from the other seven samples by their source of tissue instead of the inflammatory condition. The principal components analysis (PCA) presented a similar clustering of the samples (**Figure 1B**). The transcriptome differences between different segments of epididymis were also reported (15–17). In order to remove the confounding factors, only the caput epididymides were included for downstream analysis. After sample removal, UMAP analysis showed that the caput samples were divided mainly into two groups associated with the inflammatory condition (**Figure 1C**). As well, the correlation matrix analysis based on global transcripts revealed high correlation coefficiency (>0.97), suggesting the relatively low transcriptional heterogeneity of these samples (**Figure 1D**).

### DEGs Were Enriched in Leukocytes Chemotaxis and Ion Channel-Related Pathways

**Figure 2:**
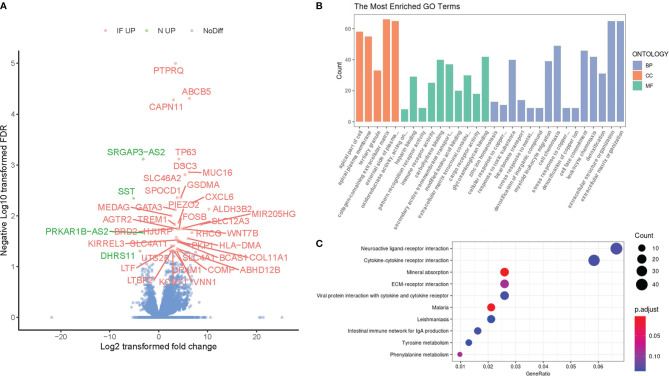
Identification of differentially expressed genes (DEGs) and their functional enrichment analysis. **(A)** The volcano plot of DEGs between chronic epididymitis (CE) and the controls with the most significant DEGs labeled and highlighted (IF UP, upregulated in CE; NC UP, upregulated in the controls; NoDiff, no significant difference). **(B)** The most enriched GO terms for the DEGs. The horizontal axis represented different GO categories: the top 15 biological processes (BP), top 5 cellular components (CC), and top 10 molecular functions (MF). *P <* 0.05 & False discovery rate (FDR) < 0.05. **(C)** The top enriched KEGG pathways for the DEGs. The horizontal axis indicated the gene ratio which was defined as the number of DEGs associated with particular term divided by the total number of the DEGs. *P <* 0.05.

We further perform a generalized linear model (GLM) by DESeq2 to compare the transcriptional profile between the inflamed caput epididymides and the controls. A total of 1727 differentially expressed genes were identified (1220 upregulated and 507 downregulated) and visualized in a volcanic plot (**Figure 2A**, *P*<0.05), with the label of 42 most significant genes (adjusted *P*<0.05). Among these identified genes, 38 transcripts were upregulated while 4 were downregulated in the inflamed caputs. Most of these genes were related to inflammation development, such as GSDMA, CXCL6, and FOSB, and some of them were involved in the fibrosis of the epididymis, such as MUC16, AGTR2, and TREM1 The six DEGs were selected for the validation with real-time PCR. The results demonstrated a significant difference of these DEGs between CE and the controls (**Supplemental Figure S2**).

To compare the overall gene reciprocal network between inflammatory and physiological status in the epididymis, gene ontology process enrichment analysis was performed based on these 1830 DEGs. The top 15 most enriched GO terms in the Biology Process, top 10 in Molecular Function, and top 5 in cellular components were shown (**Figure 2B**). Among these terms, more than three referred to leukocyte chemotaxis or extracellular milieu reconstruction, both of which were significant inflammation events in situ. Besides, ion channel and transporter-related terms were another frequent class in the enrichment results. In addition, the Kyoto Encyclopedia of Genes and Genomes (KEGG) was also performed, and the top 10 enrichment pathways were shown (**Figure 2C**). Several receptor interaction pathways, including neuroactive ligand, cytokine-cytokine, ECM, and viral protein, were essential in CE.

### GSEA Analysis Indicated the Involvement of MiRNA-Level Epigenetic Regulation in CE

**Figure 3:**
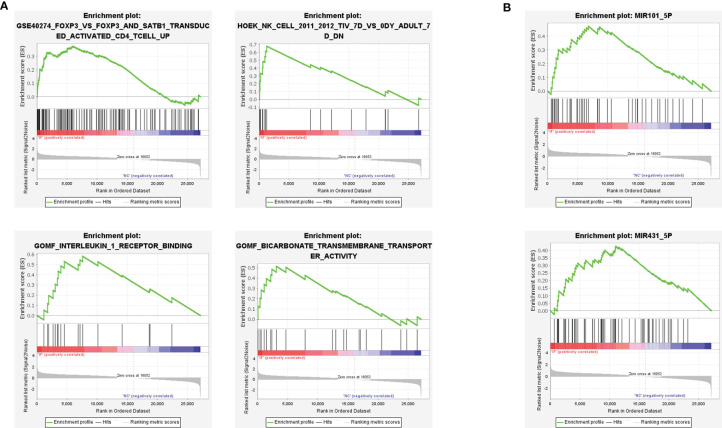
Gene set enrichment analysis (GSEA) revealed the inflammatory signatures in CE. **(A)** The immune features of gene sets were enriched in CE. *P* < 0.05. **(B)** The miRNA regulation pathway of gene sets was enriched in CE. *P* < 0.05.

To further investigate the potential inflammation progress in CE, a knowledge-based approach Gene set enrichment analysis (GSEA) was applied, and four enrichment sets were shown (**Figure 3A**). The top left set indicated the enriched genes in single Foxp3 transduced cells when compared to STAB1, a Treg signature enhancing gene, and FOXP3 simultaneously transduced cells, suggesting the less regulative immune content in CE. The top right set indicated the enrichment of NK cells, and the bottom left set showed the enhanced interleukin-1 signaling, echoing the DEGs and GO enrichment results.

Besides, two miRNA-based epigenetic sets were also determined by GSEA analysis (**Figure 3B**). Previous studies demonstrated that the miR-431-5p regulated cell proliferation and apoptosis in inflamed tissue, while miR-101-5p inhibited tumor growth by targeting the RAS gene family or CXCL6 axis (18, 19). Therefore, both of the enrichments suggested the potential small RNA-based epigenetic involvement in CE.

### Small RNA Profile Suggested Similar Heterogeneity of Samples Revealed by mRNA Expression

**Figure 4:**
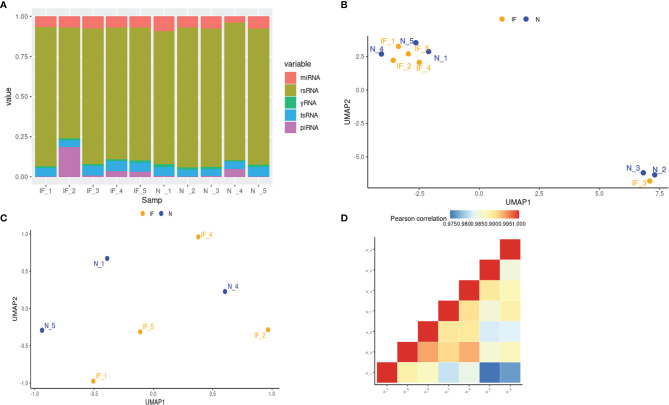
Small RNA profiles of CE and the controls. **(A)** The stacked histogram represented the distribution of small RNA species in each sample. **(B)** Visualization of 10 samples based on global miRNA expression in a two-dimension space by UMAP. IF represented the inflamed epididymides, and N indicated the controls. **(C)** Visualization of 7 samples based on global miRNA expression after removing the corpus and cauda samples in a two-dimension space by UMAP. **(D)** Global miRNA expression correlation matrix of the 7 caput epididymides.

To further investigate the change and potential impact of the epigenetic profile during inflammation in CE, small RNA sequencing for RNA fragments below 30bp length was applied. Sequencing produced 58525289 raw reads from CE and 61877245 raw reads from the controls as shown (**Supplemental Table S3**). The proportion of clean reads was >80% for each sample. These reads were then be classified to miRNA, Piwi-interacting RNA (piRNA), tsRNA, y RNA, and rRNA-derived small RNA (rsRNA) by mapping and annotation analysis. The proportion of each class of small RNAs was demonstrated for each sample, and no significant differences were observed between CE and the controls. (**Figure 4A**; **Supplemental Table S4**).

Before downstream analysis, the heterogeneity of the samples was further investigated. Similar to the transcriptional profile, the sample distance of the small RNA profile shown in UMAP indicated that the corpus and cauda have their distinctive characteristics (**Figure 4B**). To eliminate the impact of tissue heterogeneity and match the transcriptome profiling analysis, the small RNA data of two corpora and one cauda was a shield for the following analysis. Nevertheless, the small RNA profile of remained 7 samples from CE and the controls exhibited a higher heterogeneity by UMAP and correlation matrix in comparison with transcriptome level (**Figures 4C, D**), suggesting the hierarchical and complex network of small RNA regulation.

### Identification of Differentially Expressed MiRNAs (DEMs) and Functional Enrichment Analysis of Their Putative Targets

**Figure 5:**
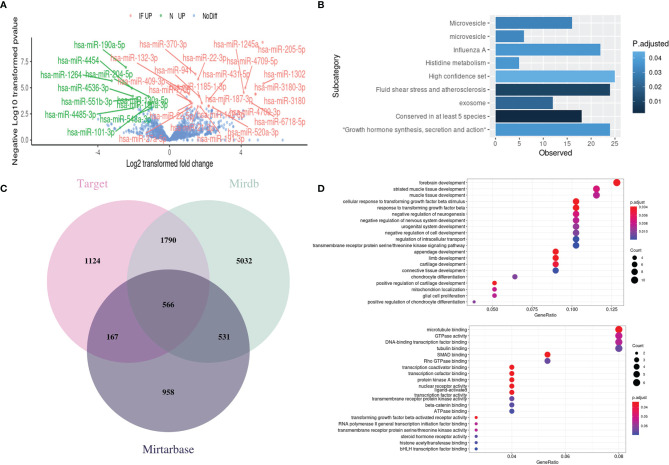
Identification of differentially expressed miRNAs (DEMs) and functional enrichment analysis of their target genes. **(A)** Volcano plot of DEMs between CE and the controls with the significant ones labeled and highlighted (IF UP, upregulated in inflamed CE; NC UP, upregulated in controls; NoDiff, no significant difference). **(B)** The enrichment plot of DEMs showed the top enriched miRNA clusters through miEAA online tools. **(C)** Venn diagram of targeted genes from the different miRNA-target prediction databases. **(D)** The top enriched GO terms in Biology Progress (top) and Molecular Function (bottom) for the putative target genes. The Vertical axis presents the GO categories, the horizontal axis represented the gene ratio which was defined as the number of the putative target genes associated with particular term divided by the total number of the putative target genes. *P* < 0.05.

We then perform GLM to detect DEMs between CE and control. A total of 34 DEMs was detected and labeled (**Figure 5A**). Specifically, 23 miRNAs were upregulated in CE while 11 miRNAs were upregulated in controls. To investigate the potential regulation of these miRNAs in chronic inflammation, the online miRNA Enrichment Analysis and Annotation Tool (miEAA) was applied. The enrichment indicates the involvement of microvesicle and influenza A related network, as well as growth hormone synthesis, secretion, and action (**Figure 5B**).

The miRNAs exert their functions by silencing the targeted mRNAs to regulate post-transcriptional protein accumulation. Therefore, we predict gene targets of 34 DEMs through the online miRNA targets prediction database miRWalk (20) to discover the potential miRNA-regulated pathway in CE. A total of 3647, 7919, and 2222 targeted genes were predicted from database Target, Mirdb, and Mirtarbase, respectively (**Figure 5C**). By intersecting the genes that were predicted at least in two databases, we get 3054 predicted genes. GO enrichment analysis was then performed on these genes, and the Top 20 terms in Biology Progress and Molecular function were shown separately (**Figure 5D**). Intriguingly, the nervous system-related pathways were highly enriched, which may help explain why the CE patients who undergo extraction surgery of epididymis in this study often suffered from pain or chronic orchalgia.

### MiRNA-mRNA Network in the Inflamed Caputs

**Figure 6:**
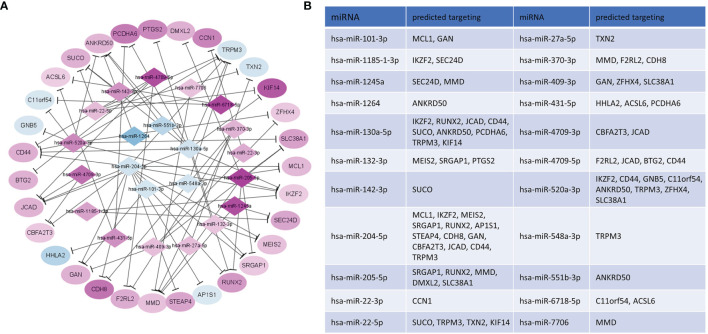
Network construction of selected potential interactions of DEM-DEG. **(A)** Purple/blue nodes represented the upregulated/downregulated mRNAs or miRNAs in CE, and the color scale of the nodes indicated the level of regulation. **(B)** Predicted target genes of DEM in CE.

To specify the epigenetic regulation network in CE, we intersected the genes predicted by miRNA targeting and detected DEGs. By matching the potential targeting pairs of miRNA and transcripts, 72 significant miRNA-mRNA pairs were identified, consisting of 22 miRNAs and 31 mRNAs (**Figure 6**). In the targeting network, 16 miRNAs were upregulated, and 6 miRNAs were downregulated; meanwhile, 25 genes were upregulated, and 5 genes were downregulated in CE. Interestingly, the hsa-miR-431-5p enriched by the GSEA analysis of DEGs was also present in this network, further supporting the miRNA-mRNA axis regulation of CE.

### The Expression Profile of TsRNAs and PiRNAs in the Inflamed Caputs

**Figure 7:**
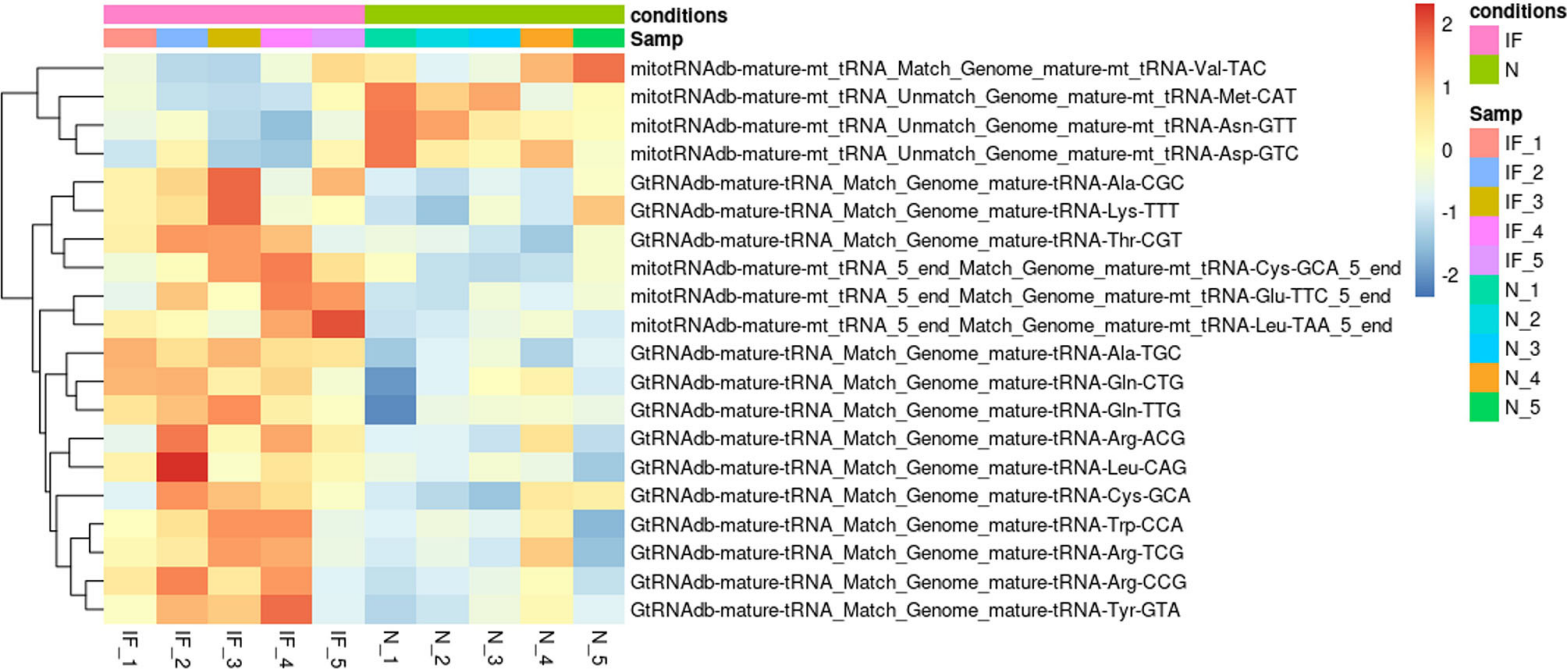
Distinct tsRNA expression pattern in CE and the controls. Heatmap plot showed the expression pattern of the top 20 distinct tsRNAs. The Vertical axis indicated the precursor of tsRNAs. *P* > 0.05. IF represented the inflamed epididymides, and N indicated the controls.

We further investigated the tsRNA profile between CE and the controls, as tsRNA was reported to be involved in inflammation and epigenetically transgenerational transmission. Although no significant difference of tsRNA species was identified, the altered pattern could be observed for the identified tsRNAs and might associated with CE (**Figure 7**). The fragments of Alanine, Glutamine, Arginine, and Leucine transfer RNA (tRNA) were highly expressed in CE, while the fragments of Aspartic, Asparagine, Methionine, and Valine tRNA were upregulated in the controls.

In addition, we also compared the piRNA profile between CE and the controls and two differentially expressed piRNAs were found (**Supplemental Materials**). The piR-has-32580 was upregulated in CE, which was predicted to related with autophagy gene ATG16L1 by online tools piRBase (21). piR-has-8721 was downregulated in CE. Other piRNAs failed to show significance between CE and the controls.

## Discussion

Based on small RNA-seq data and mRNA-seq data of CE and controls, we conducted the first study, to the best of our knowledge, for profiling the transcriptome and small RNA landscape in CE. The coalesced analysis thus provided close insight into the function of a small-RNA-based epigenetic factor in the development of CE. We totally identified 1727 DEGs and 34 DEMs between CE and the controls, which allowed us to generate a predicted interaction network consisting of 31 genes and 11 miRNAs. For the DEGs, the results showed significant enrichment of the immunoregulatory process and inflammation-related genes such as GSDMA, CXCL6, and FOSB, as well as genes of fibrosis such as MUC16, AGTR2, TREM1. Previous study of epididymitis by rat model with LPS stimulation also demonstrated the upregulated expression of CXCL6 (13). We also found the elevated expression of immune cell markers and inflammatory cytokines in CE. One of the DC markers CD209 was elevated in CE, which was coincident with our previous study of the immunohistochemical staining (22). Moreover, the expression level of IL-6 and IL-10 was upregulated 30-fold and 5-fold in CE compared with the controls, which was also proved to be the epididymitis characteristics through mouse model induced by Gram-negative (LPS) and Gram-positvie (LTA) stimulation (23). Interestingly, some DEGs in murine model such as IL-1, CCL2 was not identified in our study, suggesting the species variation or different epididymitis models and emphasizing the importance of collecting clinic samples.

Regarding the overall miRNA profile and transcriptome, our UMAP and PCA plot demonstrated that caput samples cluster together, regardless of the inflammatory or physiological condition, while corpus and cauda samples were grouped into separate clusters (**Figures 1A, B**, **4B**). This result was consistent with previous studies for mRNA profile and miRNA across epididymis, indicating that tissue heterogeneity was the main source of sample differences in the epididymis and should be considered thoroughly before analysis (7, 24, 25). We have compared the transcriptome and miRNA profile between CE and the controls after elimination of tissue specificity and produced totally different DEGs and DEMs. The enrichments of these DE features mainly fall into ERK signaling, pain-related nervous system, and kidney development, which are probably due to the specific function of the epididymis subarea.

The expression profiles of mRNA and miRNA in human CE have not been well-defined in the past. In this study, we demonstrated for the first time to obtain the mRNA and miRNA profiles in human CE. By integrating the DEGs and DEMs, we totally determined 72 pairs of miRNA-mRNA interactions (**Figure 6**), many of which were involved in inflammation and male infertility. Specifically, miR-431-5p, as upregulated and enriched by GESA analysis of DEGs in CE group, can regulate blood pressure, cell proliferation, apoptosis and epithelial-to-mesenchymal transition and the aberrant expression is associated with a different type of inflammation (26–28). HHLA2 is the target of miR-431-5p and was downregulated in CE. It encodes surface proteins on monocytes regulating cell-mediated immunity. In the past few years, as an immune checkpoint protein, the HHLA2 has aroused great interest in immunotherapy (29). the expression of PTGS2, a key enzyme in prostaglandin biosynthesis, was negatively correlated with mir-130a-5p. A similar pattern was found in the chronic constriction injury (CCI) mouse model (30). Although only female Ptgs2 knockout mice suffer from reduced fertility at multiple levels instead of male mice, it is still worthy of investigating the Ptgs2 function in male fertility due to the species differences between mouse and human (31, 32). The downregulation of TRPM3, a channel mediating calcium and sodium entry protein, might be the result of regulation by more than five miRNA species, two of which have accordant changing while the other three have opposite trending (**Figure 6**), indicating that epigenetic regulations, especially the miRNA involving, are sometimes crosslinked and complicated. TRPM3 is involved in the regulation of inflammation through the release of neuropeptides (33). Intriguingly, a study profiled the global DNA methylation in sperm and found the aberrant modification of TRPM3 in patients with low morphology scores (34). Meanwhile, miR-22-5p, the impresser of TRPM3, was found to be upregulated in testicular tissue from patients with the non-obstructive azoospermia (35). These suggest that the epigenetic regulation in the inflammatory process of CE might also have an impact on reproductive ability. TXN2 is another target of miR-22-5p and was also downregulated in CE. It was reported that the expression level of TXN2 is reduced in macrophages stimulated by LPS, and the overexpression of TXN2 can significantly attenuate interleukin-6 (IL-6) and tumor necrosis factor-alpha (TNF-α) production through NF-κB and MAPK signaling pathway (36). In addition, TXN2 can also be potentially regulated by the miR-27a-5p axis, a dual inflammation regulator in different inflammatory diseases (37, 38). These suggest the potential anti-inflammation of TXN2 in CE.

The gene products might mutually regulate the miRNA repertory. CD44 has been implicated, together with its ligand hyaluronan (HA), in several inflammatory diseases (39). In CE, CD44 is upregulated, while most of the miRNA targeting CD44 is downregulated, including miR-1264, miR-130a-5p, and miR-204-5p. The miR-1264 was reported to be suppressed by inflammatory cytokines such as TNF-a and IGF-1 (40). The miR-130a-5p and miR-204-5 are anti-inflammatory elements that act through CXCL12 and IL-6, respectively (41, 42). The synergetic variation of these functional proteins and related miRNA suggests the overall pro-inflammation milieu of CE.

The specific pattern of tsRNA in CE might be involved in inflammation development and impact male fertility, although there was no significant difference compared with the controls (**Figure 7**). The substantial effect of tsRNA on embryonic development and transgenerational transmission *via* sperm transmitting has aroused intense interest in recent years (43). However, there are few studies have investigated the relationship between epididymal inflammation and tsRNA on offspring phenotypes. Therefore, the role of tsRNA medications in the network consisting of chronic inflammation, epigenetic regulation, and offspring phenotypes remains to be further explored. piRNAs can interact with PIWI proteins and widely involved in fibrogenesis and spermatogenesis. Only two differentiated expressed piRNAs were detected in our study. Yan Li et al. also detected piR-has-32580 in normal epididymis (44). The other differentiated expressed piR-has-8721 has also been detected in testis by Angélique Girard et al, although the related function annotation is limited (45). We further examined the fibrogenesis related piRNAs like piR-823 (46) and piwi-proteins, which are involved in spermatogenesis, between two group, all of these small RNA and protein mRNA are not to be found differentially expressed. The wide variation of detected piRNA mass, from 204 to 17918, among all samples might resulted in the undetectable differentiations. To better determine the piRNA profile changing during chronic inflammation in epididymis, piRNA specific purification and sequencing should be applied during library construction (47).

Our study profiled the general mRNA and small RNA content in the epididymis and identified the segment specificity inside epididymis. However, even the caput is constituted by a bunch of heterogeneous cells and can be diverse from person to person. Shih-Hsing Leir et al. applying single cell RNA sequencing (scRNA-seq) on seven human epididymitis from testicular cancer patient. There is remarkable diversity in the structure of these epididymis as well as cell clustering. Two of donors present much higher spermatozoa population than others, suggesting the significant variation of gene expression can be accounted in bulk-RNA sequencing (48). Despite the emergent single cell study on animal and human epididymis in recent years (49, 50), relative study on epididymitis is limited. Regarding the considerable changes of immune microenvironment during CE, more refined technology such as scRNA-seq, spatial transcriptome and *in situ* transcriptome map will be required to investigate the physiological and pathological changes in CE. Taken together, our research has provided a framework for the potential involvement of small RNAs in CE and the corresponding miRNA-mRNA axis, giving a set of clues for further investigation in the immunopathological mechanisms of CE and male infertility.

## Data Availability Statement

The datasets presented in this study are deposited in the NCBI GEO database repository, accession number GSE199903.

## Ethics Statement

The studies involving human participants were reviewed and approved by Daping Hospital, Army Medical University, and The University of Hong Kong - Shenzhen Hospital. The patients/participants provided their written informed consent to participate in this study.

## Author Contributions

Y-GD and DC designed this study and contributed to the research idea. JG, PW, and J-CL contributed to the research data and wrote this manuscript. J-CL, Q-XZ, CY, YL, and DY revised and edited this manuscript. Y-GD was the guarantor of this work, and as such, had full access to all the data in the study and takes responsibility for the integrity of the data and the accuracy of the data analysis. All authors contributed to the article and approved the submitted version.

## Funding

This study was supported by Shenzhen Fundamental Research Program (JCYJ20180306173803232), Science, Technology and Innovation Commission of Shenzhen (GJHZ20200731095006018), High Level-Hospital Program, Health Commission of Guangdong Province, China (HKU-SZH201902006), and the Natural Science Foundation of Chongqing, China (cstc2020jcyj-msxmX0186).

## Conflict of Interest

The authors declare that the research was conducted in the absence of any commercial or financial relationships that could be construed as a potential conflict of interest.

## Publisher’s Note

All claims expressed in this article are solely those of the authors and do not necessarily represent those of their affiliated organizations, or those of the publisher, the editors and the reviewers. Any product that may be evaluated in this article, or claim that may be made by its manufacturer, is not guaranteed or endorsed by the publisher.
