# Supplementary material for: Integrative Analysis of Small RNA and mRNA Expression Profiles Identifies Signatures Associated With Chronic Epididymitis
Source: Front Immunol. 2022 May 11;13:883803. doi: 10.3389/fimmu.2022.883803 (PMC9130659; doi:10.3389/fimmu.2022.883803)
Supplement: Supplementary file 1 [file DataSheet_1.zip › Supplementary material/Supplementary material.docx]

Supplementary Material

# Supplementary Figures


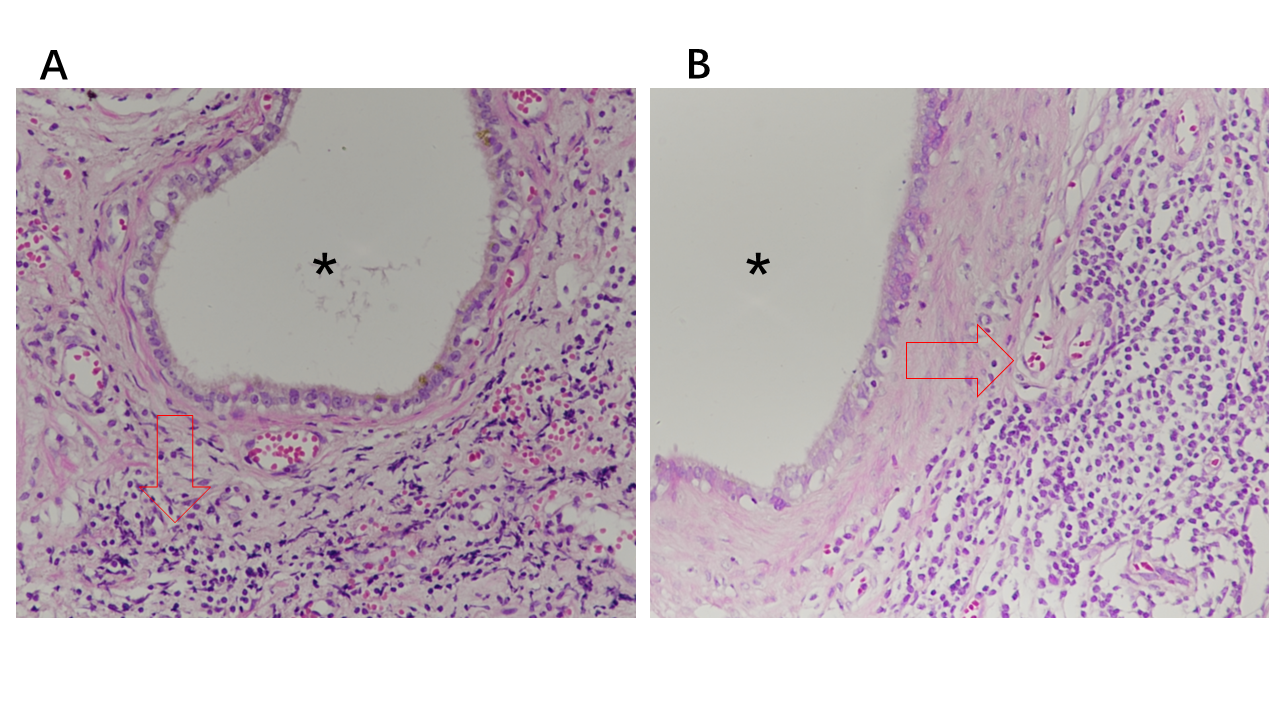
**Supplemental Figure S1**: Microphotographs of hematoxylin and eosin staining in chronic epididymitis (CE). In inflamed epididymis, the histopathological changes were characterized by monoculear cell infiltration in the interstitial and epididymal fibrosis of the CE samples. *indicates the epididymal tubule.


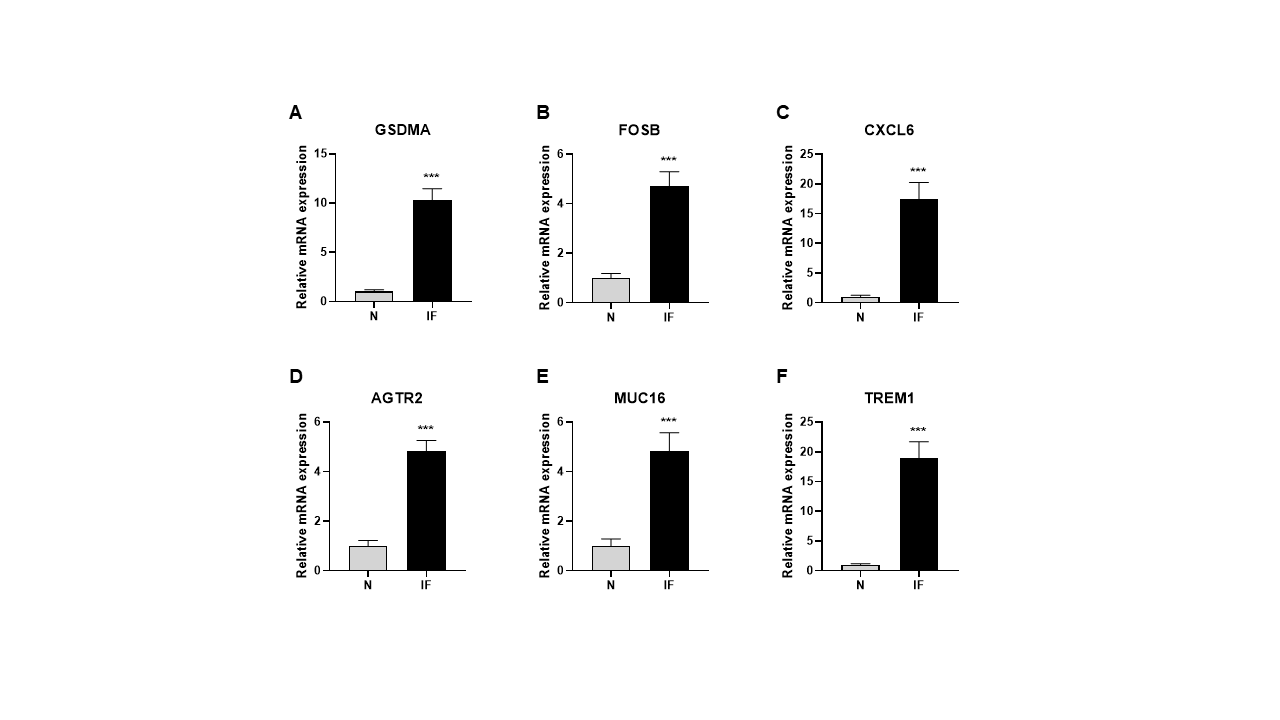


**Supplementary Figure S2**: Relative mRNA expression of 6 differentially expressed genes measured by real-time PCR. A,B,C: GSDMA, FOSB, CXCL6 involved in the inflammation of the epididymides. D,E, F: AGTR2, MUC16, TREM1 assciated with fibrosis of the epididymides. **P*<0.05, ***P*<0.01, and ****P*<0.001 in chronic epididymitis (IF, *n*=4) compared with the controls (N, *n*=3). Error bars were expressed as the standard error of the mean (SEM).

# Supplementary Tables

**Supplemental Table S1. Characteristics of chronic epididymitis patients and the controls**

| **Patient No.** | **Age (years)** | **Reproductive**  **history** | **Diagnosis** | **Course** | **Treatment** | **Pathological diagnosis** |
| --- | --- | --- | --- | --- | --- | --- |
| IF 1 | 75 | 3 daughters  1 son | 1. Chronic epididymitis (bilateral)  2. Hydrocele (right)  3. Benign prostatic hyperplasia | 1 year | 1. Epididymectomy (bilateral)  2. Hydrocelectomy (right)  3. Transurethral vapor resection of the prostate | 1. Fibrous hyperplasia of bilateral epididymides, histiocytic disorders with small lymphocyte infiltration; no granuloma and necrosis  2. Chronic inflammation of the right tunica vaginalis  3. Benign type of prostate enlargement caused by an increase number of normal prostate cells |
| IF 2 | 44 | 1 daughter | 1. Testicular trauma (left) | 1 month | 1. Orchiectomy (left) | The left testis and epididymis demonstrated typical characteristics of the traumatic lesions: local hyperemia, edema, and hemorrhage, fibrosis in some areas with acute and chronic infiltration of inflammatory cells |
| IF 3 | 53 | 1 son | 1. Chronic epididymo-orchitis (right)  2. Hydrocele (right) | 2 years | 1. Orchiectomy (right) | The right testis and epididymis demonstrated edema and chronic inflammation. |
| IF 4 | 76 | 2 daughters | 1. Chronic epididymitis (right)  2. Indirect inguinal hernia (right)  3. Benign prostatic hyperplasia | 2 years | 1. Epididymectomy (right)  2. Transurethral vapor resection of the prostate | 1. The right epididymis demonstrated interstitial hemorrhage and lymphocyte infiltration  2. Benign hyperplasia of the prostate |
| IF 5 | 72 | 2 sons | 1. Chronic epididymitis (right)  2. Benign prostatic hyperplasia | 1 year | 1. Epididymectomy (right)  2. Transurethral vapor resection of the prostate | 1. The right epididymis demonstrated local congestion and hemorrhage, fibrosis in some areas with chronic infiltration of inflammatory cells  2. Benign hyperplasia of the prostate |
| N 1 | 68 | 1 daughter  2 sons | 1. Prostate cancer with bone metastases  2. Benign prostatic hyperplasia | 1 year | 1. Orchiectomy (bilateral) | Degenerative changes of bilateral testes and epididymides, and absence of cancer cells and inflammation |
| N 2 | 57 | 2 daughters | 1. Prostate cancer with bone metastases  2. Type 2 diabetes | 1.5 years | 1. Orchiectomy (bilateral) | Normal structure of bilateral testes and epididymides, and absence of cancer cells |
| N 3 | 83 | 1 daughter  2 sons | 1. Prostate cancer with bone metastases  2. Hypertension | 2 years | 1. Orchiectomy (bilateral) | Advance degenerative changes of bilateral testes and epididymides, and absence of cancer cells |
| N 4 | 75 | 2 daughters  2 sons | 1. Prostate cancer with bone metastases  2. Acute urinary retention | 2 years | 1. Orchiectomy (bilateral) | Degenerative changes of bilateral testes and epididymides, and absence of cancer cells and inflammation |
| N 5 | 78 | 2 sons | 1. Prostate cancer with systemic metastases  2. Chronic bronchitis | 1 year | 1. Orchiectomy (bilateral) | Normal structure of bilateral testes and epididymides, and atrophic changes caused by aging |

IF represents the inflamed caput epididymides, and N indicates the controls.

**Supplemental Table S2.** Quality control and mapping statistics of mRNA reads.

| Sample | Raw reads number | Clean reads number | clean rate(%) | Mapped reads | Uniquely mapped reads | Multiple mapped reads |
| --- | --- | --- | --- | --- | --- | --- |
| IF_1 | 47571972 | 46358674 | 97.37 | 43854413(94.60%) | 42084455(95.96%) | 1769958(4.04%) |
| IF_2 | 48557504 | 46984966 | 96.68 | 45626781(97.11%) | 44412322(97.34%) | 1214459(2.66%) |
| IF_3 | 42959874 | 41403086 | 96.3 | 40126282(96.92%) | 38630165(96.27%) | 1496117(3.73%) |
| IF_4 | 47035170 | 44861154 | 95.3 | 43076514(96.02%) | 41921327(97.32%) | 1155187(2.68%) |
| IF_5 | 48934226 | 46084566 | 93.78 | 43912140(95.29%) | 42405992(96.57%) | 1506148(3.43%) |
| N_1 | 49315688 | 46981478 | 95.14 | 45024696(95.83%) | 43767882(97.21%) | 1256814(2.79%) |
| N_2 | 46314478 | 44057432 | 95.05 | 42127108(95.62%) | 40957466(97.22%) | 1169642(2.78%) |
| N_3 | 49393064 | 47922790 | 96.98 | 46490655(97.01%) | 45231065(97.29%) | 1259590(2.71%) |
| N_4 | 48494710 | 46268198 | 95.34 | 44353811(95.86%) | 42769188(96.43%) | 1584623(3.57%) |
| N_5 | 47369016 | 45410270 | 95.78 | 43649433(96.12%) | 42343183(97.01%) | 1306250(2.99%) |

IF represents the inflamed caput epididymides, and N indicates the controls.

**Supplemental Table S3**. Quality control and mapping statistics of small RNA reads.

| Sample | Num. of Raw Reads | Clean Reads% | Remove Adapter% | Insert Null% | N% | Too short% | Poly-A% | Too long% | Low  quality% |
| --- | --- | --- | --- | --- | --- | --- | --- | --- | --- |
| IF_1 | 12228898 | 95.62% | 0.02% | 0.07% | 0.05% | 1.96% | 0.02% | 1.18% | 1.08% |
| IF_2 | 13095868 | 90.12% | 0.02% | 0.28% | 0.04% | 7.99% | 0.05% | 0.41% | 1.08% |
| IF_3 | 11506368 | 92.82% | 0.01% | 0.11% | 0.05% | 1.61% | 0.05% | 4.26% | 1.09% |
| IF_4 | 10393841 | 91.46% | 0.01% | 0.70% | 0.04% | 2.96% | 0.06% | 3.55% | 1.22% |
| IF_5 | 11300314 | 91.11% | 0.01% | 0.15% | 0.05% | 2.11% | 0.08% | 5.23% | 1.26% |
| N_1 | 11658280 | 96.09% | 0.01% | 0.07% | 0.04% | 2.09% | 0.04% | 0.39% | 1.27% |
| N_2 | 14417012 | 95.96% | 0.02% | 0.07% | 0.05% | 2.02% | 0.05% | 0.48% | 1.35% |
| N_3 | 14098882 | 94.76% | 0.01% | 0.10% | 0.04% | 1.29% | 0.04% | 2.70% | 1.06% |
| N_4 | 10226219 | 83.07% | 0.06% | 0.19% | 0.04% | 14.51% | 0.05% | 1.15% | 0.93% |
| N_5 | 11476852 | 86.77% | 0.01% | 0.11% | 0.04% | 1.29% | 0.08% | 10.63% | 1.07% |

IF represents the inflamed caput epididymides, and N indicates the controls.

**Supplemental Table S4.** Species distribution of the mapped small RNA reads.

|  | **miRNA** | **rsRNA** | **yRNA** | **tsRNA** | **piRNA** | **Samp** |
| --- | --- | --- | --- | --- | --- | --- |
| IF_1 | 5985.77 | 77184.66 | 960 | 4687.36 | 204.36 | distribution_IF_1 |
| IF_2 | 6892.22 | 67271.09 | 854 | 4516.05 | 17920 | distribution_IF_2 |
| IF_3 | 6002 | 66704.26 | 940 | 4692.39 | 606.36 | distribution_IF_3 |
| IF_4 | 5776.44 | 66993.66 | 894 | 5259.42 | 2813.36 | distribution_IF_4 |
| IF_5 | 5896.33 | 69730.19 | 1050 | 4836.84 | 2702.36 | distribution_IF_5 |
| N_1 | 6138.44 | 54416.88 | 1024 | 3609.18 | 434.36 | distribution_NC_1 |
| N_2 | 6769.22 | 87379.6 | 1174 | 4413.25 | 227.27 | distribution_NC_2 |
| N_3 | 6300 | 72571.61 | 960 | 3803.07 | 396.09 | distribution_NC_3 |
| N_4 | 5531 | 116855.68 | 1272 | 6142.76 | 6724.09 | distribution_NC_4 |
| N_5 | 5168.44 | 57883.72 | 996 | 3964.63 | 154.27 | distribution_NC_5 |

**Supplemental Table S5.** The primers used for real-time PCR.

| Primer | Sequence (5’ to 3’) |
| --- | --- |
| F-GSDMA | AGAACAGCACTCTGGAGGTCCA |
| R-GSDMA | CCATCACCACATACAGGTTCTCC |
| F-CXCL6 | GGGAAGCAAGTTTGTCTGGACC |
| R-CXCL6 | AAACTGCTCCGCTGAAGACTGG |
| F-FOSB | TCTGTCTTCGGTGGACTCCTTC |
| R-FOSB | GTTGCACAAGCCACTGGAGGTC |
| F-MUC16 | GATGTCAAGCCAGGCAGCACAA |
| R-MUC16 | GAGAGTGGTAGACATTTCTGGGC |
| F-AGTR2 | CCATGTTCTGACCTTCCTGGATG |
| F-AGTR2 | CGGATTAACGCAGCTGTTGGTG |
| F-TREM1 | CGATGTCTCCACTCCTGACTCT |
| R-TREM1 | CAGCAAACAGGACAGAGAAGACC |
